# Trends in nontraumatic subarachnoid hemorrhage-related mortality among young adult (15–64 years) population in the United States, 1999–2022

**DOI:** 10.3389/fneur.2025.1646709

**Published:** 2025-10-03

**Authors:** Olivia Foley, Syeda Aamna Ijtaba Rizvi, Thomas C. Kensok, Vikram Murugan, Anthony Ashby, Ali Bin Abdul Jabbar, Mohsin Mirza, Abubakar Tauseef

**Affiliations:** ^1^Creighton University School of Medicine, Omaha, NE, United States; ^2^Dow University of Health Sciences, Karachi, Pakistan; ^3^CHI Health Immanuel Medical Center, Omaha, NE, United States; ^4^Creighton University Department of Internal Medicine, Omaha, NE, United States

**Keywords:** nontraumatic subarachnoid hemorrhage, young adults, mortality, disparities, trends

## Abstract

**Background:**

Nontraumatic subarachnoid hemorrhages (NSAH) are the result of intracranial aneurysm rupture and involve bleeding into subarachnoid space. NSAH causes significant stroke burden among adults in the United States (US). Due to the unique challenges that younger adults (aged 15–64 years) may face, it is important to analyze NSAH-related mortality among this age group stratified to determine who is most at risk.

**Methods:**

Mortality trends related to NSAH in adults aged 15–64 years old in the US between 1999 and 2022 were analyzed utilizing the Centers for Disease Control and Prevention Wide-ranging Online Data for Epidemiologic Research (CDC WONDER) database. Age-adjusted mortality rate (AAMR), annual percent change (APC), and average annual percent change (AAPC) were subsequently analyzed. Data was then stratified by sex, race, region, state, rural vs. urban classification, and age.

**Results:**

Between 1999 and 2022, there were 85,930 NSAH-related deaths among adults aged 15–64 years in the US, and overall AAMR decreased throughout the study period. Females had consistently higher AAMRs than males but demonstrated a larger overall decrease in mortality. Black or African American and American Indian or Alaskan Native patients had the highest NSAH-related AAMR between 1999 and 2022. NSAH-related mortality decreased in all regions of the US between 1999 and 2022, while individual states demonstrated a variety of trends. Urban and rural areas both saw declines in NSAH-related AAMR, while the 55–64-year age-group consistently displayed the highest NSAH-related crude mortality rates between 1999 and 2022.

**Conclusion:**

Despite overall declines in mortality, persistent disparities in mortality across sex, race, and region highlight need for further study to decrease NSAH-related burden these groups overall.

## Introduction

Nontraumatic subarachnoid hemorrhage (NSAH) is a serious cerebrovascular event characterized by bleeding into the subarachnoid space, often due to the rupture of an intracranial aneurysm. NSAH accounts for approximately 5–10% of all strokes in the United States and remains a significant cause of morbidity and mortality despite advancements in neurosurgical and critical care management (1). While NSAH incidence is typically associated with older populations, a substantial proportion of cases occur in younger adults, defined as individuals aged 15 to 64 years (2, 3). Because many younger adults affected by NSAH are in their prime working years, they face unique challenges, including increased long-term disability, economic burden, and reduced quality of life (4).

Although studies have explored risk factors for NSAH, including hypertension, smoking, alcohol use, and genetic predisposition, research on age-specific mortality trends in younger adults remains limited (5, 6). Up until this point, specific analysis of NSAH incidence and mortality in young adults has not been performed using a national and epidemiological level in the published literature. This knowledge gap is particularly concerning as epidemiological trends suggest a rising incidence of stroke in younger adults, yet data on SAH-specific mortality patterns within this group are scarce (7, 8). Additionally, racial and socioeconomic disparities may contribute to differences in subarachnoid hemorrhage outcomes, further underscoring the need for targeted public health interventions (9, 10).

Understanding NSAH-related mortality trends in young adults is critical for improving prevention strategies, optimizing early detection, and enhancing post-hemorrhagic care. This study aims to analyze temporal trends in NSAH-related mortality among young adults aged 15–64 in the United States, with a focus on demographic variations and evolving risk factors. The Centers for Disease Control and Prevention Wide-ranging Online Data for Epidemiologic Research (CDC WONDER) database provides epidemiological data that aligns with the objective of examining national trends in NSAH incidence. CDC WONDER was chosen over other similar databases due to its national coverage and focus on a non-cancer condition (11, 12). By examining national data, this study seeks to provide insights into whether mortality rates have declined over time and to identify potential disparities that may inform future healthcare policies and stroke prevention efforts.

## Methods

### Study design

Researchers analyzed mortality data from the CDC WONDER database regarding deaths related to NSAH among young adults aged 15 to 64 years old between 1999 and 2022. The Multiple Cause of Death database from the CDC WONDER site was utilized to analyze all deaths associated with this specific disease state. The International Classification of Diseases (ICD), 10^th^ Revision, Clinical Modification codes were utilized to separate out data relevant to this study. To capture all data regarding NSAH, the ICD-10 code I60 was utilized, which includes both aneurysmal and non-aneurysmal causes of NSAH.

Data was extracted from two distinct time periods, 1999 to 2020 and 2020 to 2022, and then combined for analysis. Data regarding NSAH-related deaths was analyzed across different sexes races, regions, states, rural vs. urban settings, and age. The relevant data extracted included number of deaths, crude mortality rates, and age-adjusted mortality rates (AAMR) with 95% confidence intervals (CI) for each stratifying variable. The CDC WONDER database contains public information that is anonymized; as such, this study was exempt from institutional review board (IRB) approval.

### Study population

The study population included five age groups in 10-year intervals from 15–24, 25–34, 35–44, 45–54, and 55–64 years old. Sexes included male or female. Race and ethnicity groups included White, Black or African American, Asian or Pacific Islander, and Hispanic or Latino. Data was stratified into rural and urban classifications, based on the National Center for Health Statistics Urban–Rural Classification Scheme as used by the CDC WONDER database (13). Data was also stratified by state, as well as by United States census regions (Northeast, Midwest, South, and West), as defined by the Census Bureau definitions utilized by the CDC WONDER database. States included in each region can be seen in Table 1. Regional data as well as rural vs. urban data were only analyzed between 1999 and 2020 because data provided for 2020–2022 was marked as provisional on the CDC WONDER database.

**Table 1:** States included in United States Census Bureau Regions.

States included in United States Census Bureau Regions			
Northeast	Midwest	West	South
Connecticut	Illinois	Arizona	Delaware
Maine	Indiana	Colorado	District of Columbia
Massachusetts	Michigan	Idaho	Florida
New Hampshire	Ohio	Montana	Georgia
Rhode Island	Wisconsin	Nevada	Maryland
Vermont	Iowa	New Mexico	North Carolina
New Jersey	Kansas	Utah	South Carolina
New York	Minnesota	Wyoming	Virginia
Pennsylvania	Missouri	Alaska	West Virginia
	Nebraska	California	Alabama
	North Dakota	Hawaii	Kentucky
	South Dakota	Oregon	Mississippi
		Washington	Tennessee
			Arkansas
			Louisiana
			Oklahoma
			Texas

### Statistical analysis

AAMR as well as average annual percentage change (AAPC) were analyzed throughout the study period among each stratification. Data were compiled and organized via Microsoft Excel, where the article’s primary figures were created. Beyond initial charts and graphs, Joinpoint regression was conducted to further analyze this data. This study utilized the Joinpoint Regression Program (Joinpoint version 4.9.0.0 available via the National Cancer Institute in Bethesda, Maryland) to determine both overall mortality trends as well as those specified to stratified variables (14). Joinpoint regression allowed researchers to identify whether significant changes in mortality occurred throughout the study period and if so, when those changes occurred (14). The Monte Carlo permutation test was also utilized to calculate annual percent changes (APC) for each AAMR calculated, all reported with 95% CIs (14). Average values of the APCs were calculated as AAPCs and reported to further highlight mortality trends throughout the study period. Differences in mortality and trends were analyzed utilizing 2-tailed t-tests with statistical significance set at *p* ≤ 0.05. Statistically significant data is represented with an asterisk ‘*’ throughout this paper.

## Results

### Overall

Between 1999 and 2022 there were 85,930 deaths related to NSAH. Annual total deaths, as well as annual stratification of mortality by sex as well as by race/ethnicity can be seen in Table 2.

**Table 2:** Total NSAH deaths stratified by sex and race.

Year	Overall Deaths	Female Deaths	Male Deaths	American Indian or Alaska Native Deaths	Asian or Pacific Islander Deaths	Black or African American Deaths	White Deaths	Hispanic Deaths
1999	4,198	2,506	1,692	32	152	802	2,764	427
2000	4,253	2,629	1,624	37	151	785	2,822	441
2001	3,735	2,223	1,512	29	151	738	2,390	418
2002	3,995	2,395	1,600	32	157	772	2,588	437
2003	3,937	2,366	1,571	34	175	750	2,515	447
2004	3,972	2,351	1,621	45	162	763	2,543	450
2005	3,838	2,278	1,560	42	159	742	2,448	440
2006	3,828	2,232	1,596	21	183	727	2,417	477
2007	3,717	2,135	1,582	40	175	705	2,297	493
2008	3,463	2007	1,456	25	176	656	2,151	450
2009	3,514	2028	1,486	34	180	630	2,166	491
2010	3,395	1988	1,407	25	194	637	2065	461
2011	3,369	1954	1,415	37	196	608	2028	495
2012	3,355	1922	1,433	29	204	613	2008	491
2013	3,225	1843	1,382	31	173	562	1916	535
2014	3,197	1773	1,424	25	180	589	1889	507
2015	3,219	1878	1,341	39	196	552	1914	507
2016	3,406	1896	1,510	37	227	615	1974	541
2017	3,204	1778	1,426	33	213	575	1835	533
2018	3,252	1816	1,436	31	195	588	1853	568
2019	3,288	1879	1,409	35	209	587	1832	616
2020	3,566	1948	1,618	42	234	639	1998	648
2021	3,627	1918	1709	39	235	686	1924	704
2022	3,377	1904	1,473	42	212	624	1790	665
Total:	85,930	49,647	36,283	816	4,489	15,945	52,127	12,242

**Figure 1:**
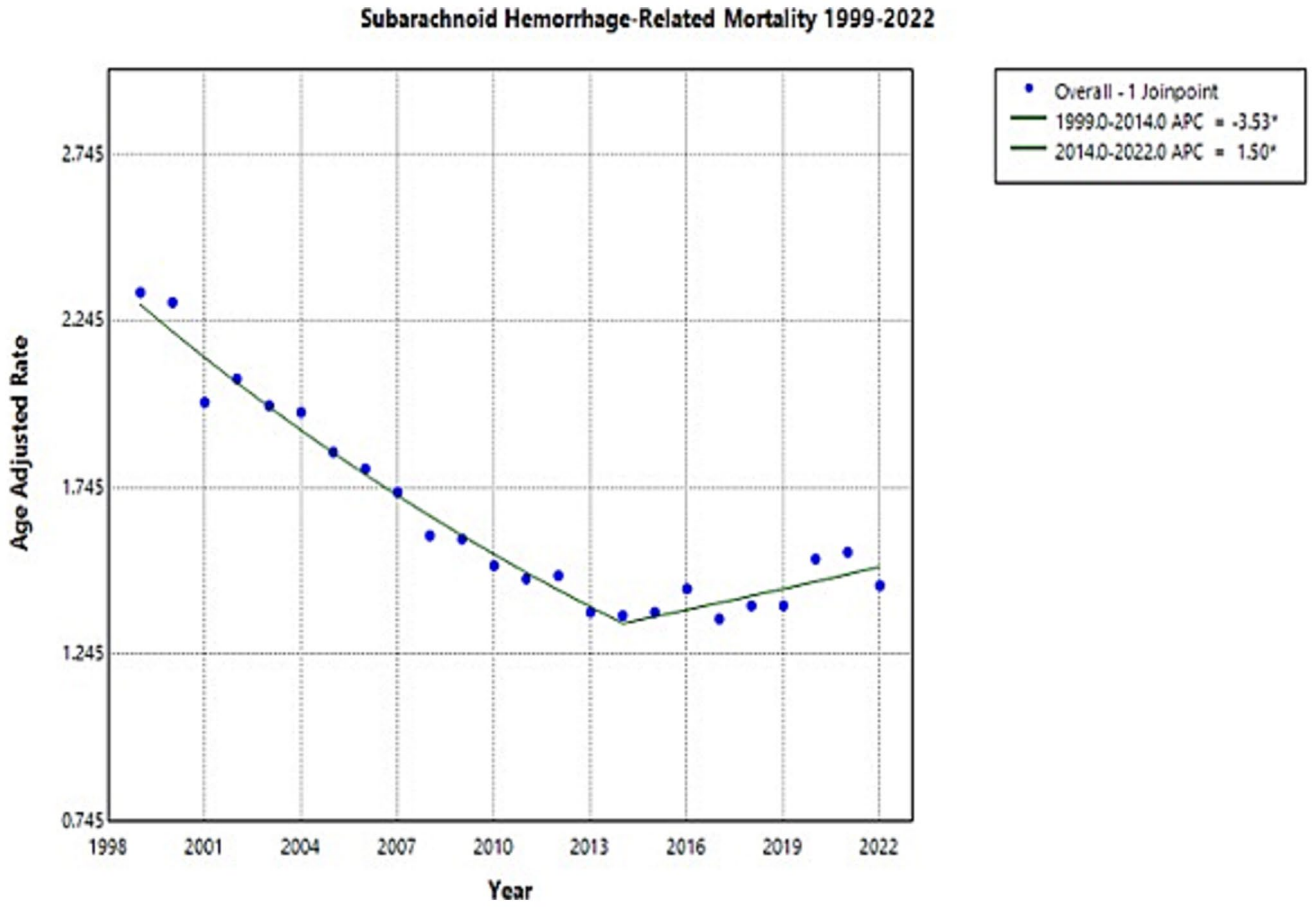
Multiple Joinpoint model of overall NSAH mortality between 1999 and 2022.

Overall, the NSAH-related AAMR decreased between 1999 and 2022 (AAPC: −1.81*, 95% CI: −2.12 to −1.55) (Figure 1; Supplementary Figure 1; Supplementary Table 1). Overall AAMR decreased from 2.33 in 1999 to 1.36 in 2014 (APC: −3.53*, 95% CI: −4.08 to −3.15), indicating a significant reduction in mortality. AAMR then increased insignificantly to 1.45 in 2022 as a tentative observation (APC: 1.50, 95% CI: 0.39 to 3.16) but still represented a downward trend throughout the study period (Figure 1; Supplementary Table 1). These short-term fluctuations require further investigation, but overall, long-term decline in NSAH-related mortality occurred between 1999 and 2022 in the US.

### Sex

**Figure 2:**
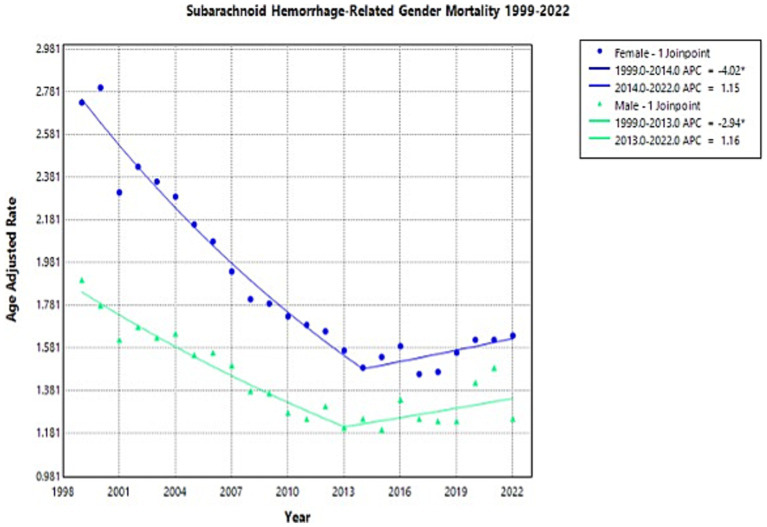
Multiple Joinpoint model of NSAH mortality stratified by sex between 1999 and 2022.

Females had consistently higher AAMRs than males throughout the study period (Supplementary Figure 1). However, females did have a larger decrease in overall mortality than men between 1999 and 2022, respectively (AAPC −2.25*, −1.36* respectively) (Supplementary Table 1; Figure 2). Men’s AAMR decreased significantly from 1.9 in 1999 to 1.21 in 2013 (APC: −2.94*, 95% CI: −4.44 to −2.20), but proceeded to increase to 1.25 in 2022, a brief temporal trend noted in this study’s results (APC: 1.16, 95% CI: −0.31 to 5.09) (Supplementary Table 1; Figure 2). Females experienced a decline in AAMR from 1999 to 2014, with the value dropping from 2.73 to 1.49 (APC: −4.02*, 95% CI: −4.52 to −3.61) (Supplementary Table 1; Figure 2). Female AAMR proceeded to increase to 1.64 in 2022, however, which is a tentative observation that must be further evaluated (APC: 1.15, 95% CI: −0.09 to 3.14) (Supplementary Table 1; Figure 2).

### Race

Throughout the study, there was wide variation in NSAH-related AAMR across different racial groups, with Black or African American patients and American Indian or Alaskan Native patients having the highest AAMRs overall (Supplementary Figure 2).

**Figure 3:**
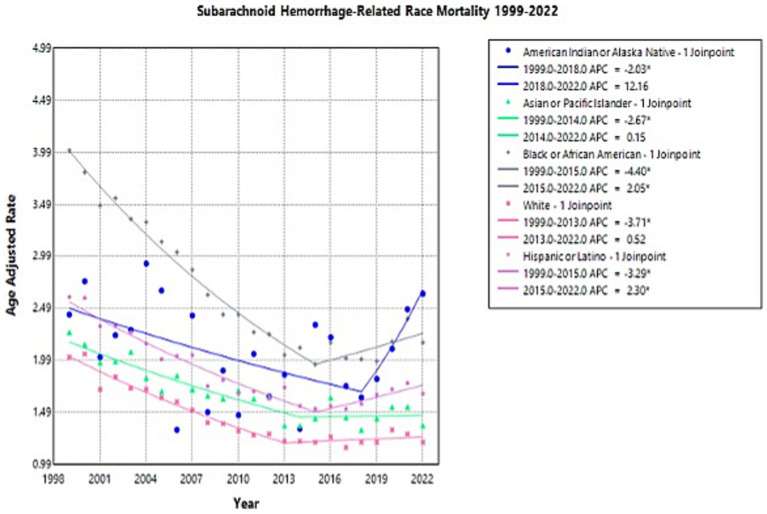
Multiple Joinpoint model of NSAH mortality stratified by race between 1999 and 2022.

American Indian or Alaska Native patients experienced a decrease in AAMR from 2.43 in 1999 to 1.63 in 2018 (APC: −2.03*, 95% CI: −8.42 to −0.47), but a short-term rise to 2.63 in 2022 (APC: 12.16, 95% CI: −0.23 to 40.73) (Supplementary Table 2; Figure 3). This represents an overall increase in NSAH-related AAMR among Native American patients between 1999 and 2022, although shorter term observations must be further analyzed (AAPC: 0.30, 95% CI: −1.72 to 1.79) (Supplementary Table 2; Figure 3).

Asian or Pacific Islander NSAH patients experienced a decrease in AAMR from 2.26 in 1999 to 1.36 in 2014 (APC: −2.67*, 95% CI: −7.33 to −1.22) but a subsequent increase to 1.36 in 2022 (APC: 0.15, 95% CI: −1.86 to 7.60) (Supplementary Table 2; Figure 3). This represents an overall decrease in NSAH-related AAMR among Asian or Pacific Islander patients between 1999 and 2022, with some short-interval fluctuations throughout the study period (AAPC: −1.69*, 95% CI: −2.40 to −1.01) (Supplementary Table 2; Figure 3).

Black or African American NSAH patients experienced a decrease in AAMR from 4.01 in 1999 to 1.95 in 2015 (APC: −4.40*, 95% CI: −5.03 to −3.92) but a subsequent increase to 2.16 in 2022 (APC: 2.05*, 95% CI: 0.36 to 5.30) (Supplementary Table 2; Figure 3). This represents an overall decrease in NSAH-related AAMR among Black or African American patients between 1999 and 2022, with short-term fluctuations that require further analysis (AAPC: −2.48*, 95% CI: −2.86 to −2.14) (Supplementary Table 2; Figure 3).

White patients experienced a decrease in AAMR from 2.02 in 1999 to 1.21 in 2013 (APC: −3.71*, 95% CI: −4.33 to −3.23), followed by little change in AAMR at 1.20 in 2022 (APC: 0.52, 95% CI: −0.50 to 2.34) (Supplementary Table 2; Figure 3). This represents an overall decrease in NSAH-related AAMR among White patients between 1999 and 2022, with outside factors potentially contributing to the tentative trends found within the study period (AAPC: −2.07*, 95% CI: −2.38 to −1.79) (Supplementary Table 2; Figure 3).

Hispanic or Latino NSAH patients experienced a short-term decrease in AAMR from 2.60 in 1999 to 1.52 in 2015 (APC: −3.29*, 95% CI: −3.81 to −2.86) followed by a short-term increase to 1.67 in 2022 (APC: 2.30*, 95% CI: 1.01 to 4.31) (Supplementary Table 2; Figure 3). This represents an overall decrease in NSAH-related AAMR among Hispanic or Latino patients between 1999 and 2022, with short-term fluctuations throughout the study period (AAPC: −1.62*, 95% CI: −1.91 to −1.33) (Supplementary Table 2; Figure 3).

### Region

**Figure 4:**
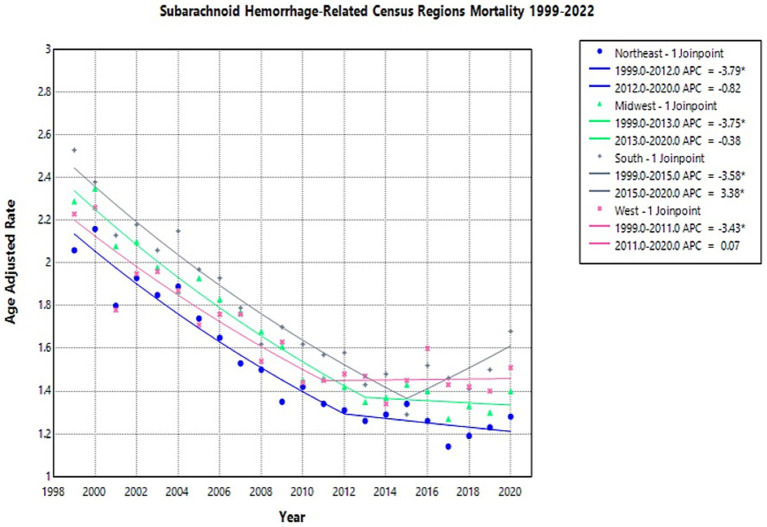
Multiple Joinpoint model of NSAH mortality stratified by US Census Region between 1999 and 2022.

Through 1999 to 2020, deaths caused by NSAH in the young-adult population decreased significantly in all regions, with AAPCs of −2.67 (CI 95%: −3.11, −2.24) in Northeast, −2.64 (CI 95%: −3.13, −2.25) in Midwest, −1.9649 (CI 95%: −2.51, −1.54) in South, −1.9426 (CI 95%: −2.45, −1.43) in West, respectively, (Figure 4; Supplementary Figure 3; Supplementary Table 3).

In 1999, the highest AAMR was observed in the South (2.53 deaths per 100,000 people), while the lowest was in the Northeast (2.06 deaths per 100,000 people) (Supplementary Table 3). The Northeast region AAMR tentatively decreased from 2.06 in 1999 to 1.31 in 2012 (APC: −3.79*, 95% CI: −5.01 to −3.19) and short-term decreased again to 1.28 in 2020 (APC: −0.82*, 95% CI: −2.21 to 2.89) (Figure 4; Supplementary Table 3). The Northeast saw a significant decrease in AAMR from 1999 to 2012, but the short-term fluctuations leveled off between 2012 and 2020 (Figure 4; Supplementary Table 3).

The Midwest region AAMR temporarily decreased from 2.29 in 1999 to 1.35 in 2013 (APC: −3.75*, 95% CI: −5.17 to −3.22) and decreased in the short-term again to 1.40 in 2020 (APC: −0.38*, 95% CI: −2.20 to 5.27) (Figure 4; Supplementary Table 3). These fluctuations represented an overall decrease throughout the study period (Figure 4; Supplementary Table 3).

The South region AAMR decreased from 2.53 in 1999 to 1.29 in 2015 (APC: −3.5777*, 95% CI: −4.44 to −3.03) and then increased to 1.68 in 2020 (APC: 3.38*, 95% CI: 0.15 to 12.22) (Figure 4; Supplementary Table 3). These represent tentative observations in the data, and represent an overall decrease in NSAH-related mortality (Figure 4; Supplementary Table 3).

The West region AAMR tentatively decreased from 2.23 in 1999 to 1.45 in 2011 (APC: −3.43*, 95% CI: −5.37 to −2.54), and saw a short-term increase to 1.51 in 2020 (APC: 0.07*, 95% CI: −1.26 to 4.13) (Figure 4; Supplementary Table 3). The West, similar to the Northeast and saw an overall decrease in NSAH-related mortality, despite short-term fluctuations throughout the study period (Figure 4; Supplementary Table 3).

### State

From 1999 to 2022, most of U.S. states experienced a significant decline in AAMRs from NSAH. States like California, Arizona, and Alabama demonstrated some of the most dramatic improvements. For instance, California’s AAMR dropped from 2.37 per 100,000 in 1999 to 0.08 in 2022, while Arizona went from 2.17 to 0.17, and Alabama from 2.88 to 0.22. These represent sustained and substantial public health gains.

Similarly, states like Georgia, Michigan, and Florida also showed consistent downward trends over the 23-year period. Florida’s AAMR, for example, decreased from 2.46 in 1999 to 0.11 in 2022, despite a temporary uptick to 2.01 in 2020.

However, not all states followed a steady path. Several, such as Hawaii, Indiana, and Mississippi, saw intermediate spikes in AAMRs. These changes represent tentative observations, those of which need further investigation to establish as true trends. Hawaii’s rate rose from 2.53 in 1999 to 3.78 in 2020 before plunging to just 0.06 in 2022. A similar trend occurred in Indiana, where AAMR rose from 1.49 to 2.14 between 1999 and 2020, then fell sharply to 0.17 by 2022. Several states—including Alaska, Montana, Rhode Island, South Dakota, North Dakota, District of Columbia and Wyoming—had missing or unreliable data in multiple years. This limits the ability to evaluate trends in those locations with confidence. State-level data in NSAH-related mortality is represented in Supplementary Figures 4, 5.

### Rural vs. urban

The AAMR for NSAH showed a general decline in both urban and rural areas from 1999 to 2020 (Supplementary Figure 6).

**Figure 5:**
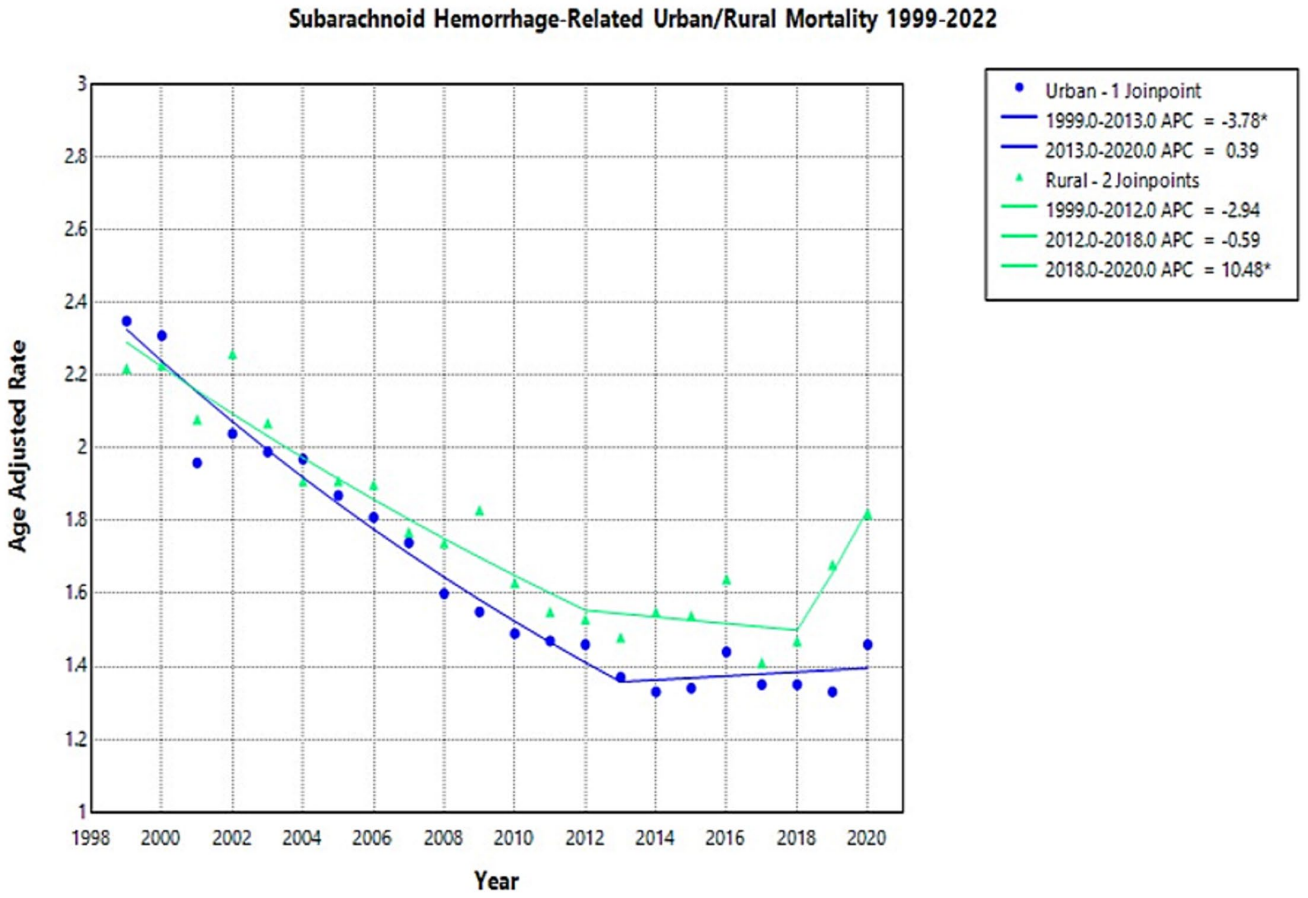
Multiple Joinpoint model of NSAH mortality stratified by rural vs. urban classification between 1999 and 2022.

In urban areas, the NSAH-related AAMR temporarily decreased from 2.35 deaths per 100,000 people in 1999 to 1.37 in 2013 (APC: −3.78*, 95% CI: −4.44 to −3.30), and then increased in the short term to 1.46 in 2020 (APC: 0.39, 95% CI: −1.11 to 3.83) (Supplementary Table 4; Figure 5). Similarly, rural areas experienced an initial tentative decline in NSAH-related AAMR from 2.22 in 1999 to 1.53 in 2012 (APC: -2.94, 95% CI: −6.45 to 1.15) (Supplementary Table 4; Figure 5). Rural areas proceeded to see a subsequent short-term decrease in NSAH-related AAMR to 1.47 in 2018 (APC: -0.59, 95% CI: −5.33 to 1.44) but then saw a tentative increase to 1.82 in 2020 (APC: 10.48*, 95% CI: 1.46 to 16.81) (Supplementary Table 4; Figure 5).

### Age

The crude mortality rate for NSAH showed a declining trend across all age groups from 1999 to 2022, though the degree of reduction varied by age (Supplementary Figure 7). The 55–64 age group consistently had the highest crude mortality rates throughout the study period (Supplementary Figure 7).

**Figure 6:**
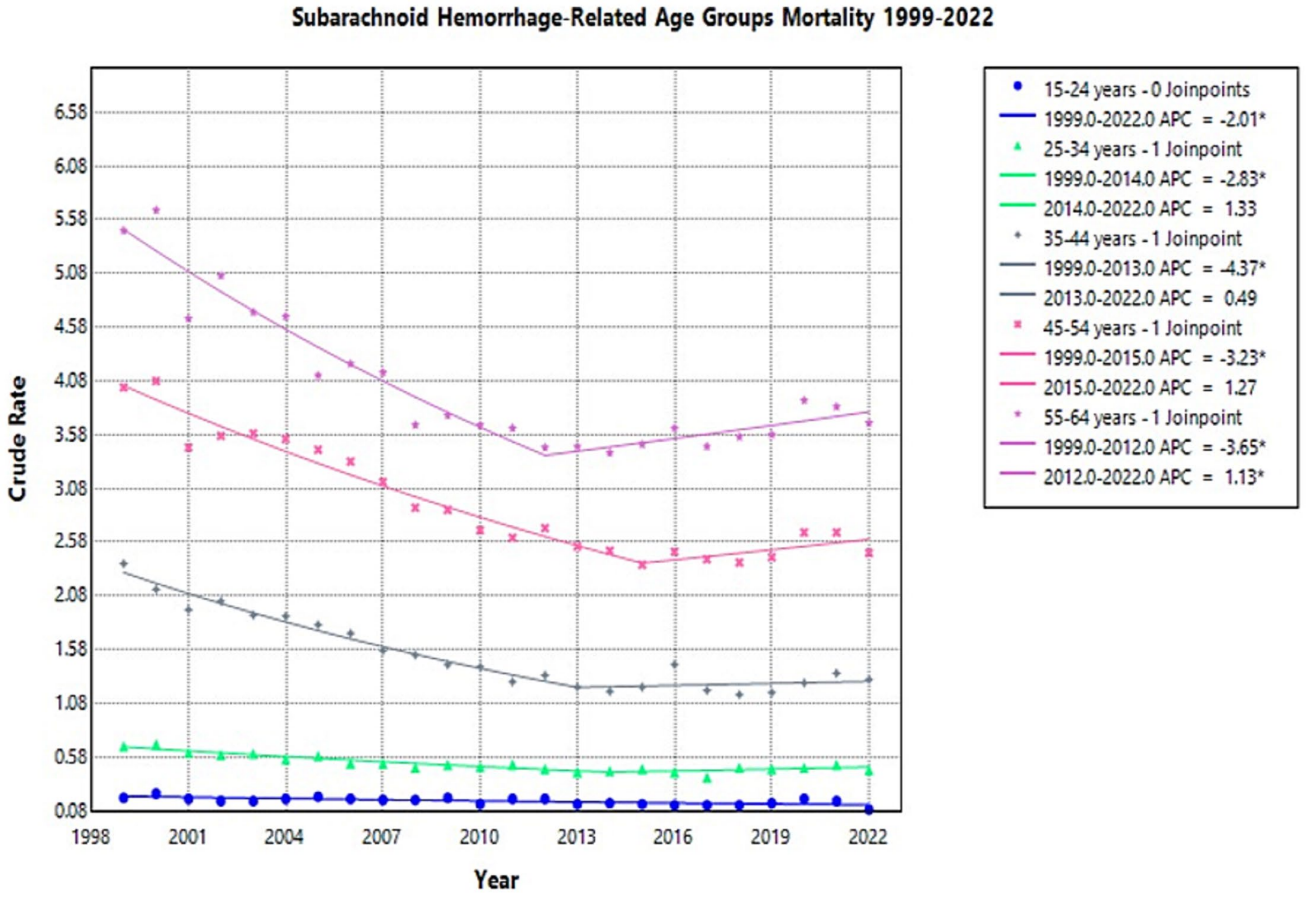
Multiple Joinpoint model of NSAH mortality stratified by 10-year age groups between 1999 and 2022.

In the 15–24 age group, the crude mortality rate remained relatively low throughout the study period but declined from 0.2 per 100,000 in 1999 to 0.09 in 2022 (APC: −2.01*, 95% CI: −3.06 to −1.06) (Figure 6; Supplementary Table 5).

For individuals aged 25–34 years, the crude mortality rate initially decreased from 0.68 in 1999 to 0.45 in 2014 (APC: −2.83*, 95% CI: −3.97 to −2.20) and then increased slightly in the short term to 0.46 in 2022 (APC: 1.33, 95% CI: −0.30 to 5.15) (Figure 6; Supplementary Table 5). Among those 35–44 years old, the crude mortality rate tentatively decreased from 2.38 in 1999 to 1.23 in 2013 (APC: −4.37*, 95% CI: −5.46 to −3.67), but then increased slightly in the short term to 1.3 in 2022 (APC: 0.49, 95% CI: −1.04 to 3.92) (Figure 6; Supplementary Table 5).

In the 45–54 age group, the crude mortality rate tentatively declined from 4.02 in 1999 to 2.37 in 2015 (APC: −3.23*, 95% CI: −4.15 to −2.72), then saw a short-term increase to 2.48 in 2022 (APC: 1.27, 95% CI: −0.79 to 8.04) (Figure 6; Supplementary Table 5). For the 55–64 age group, the crude mortality rate tentatively declined from 5.48 in 1999 to 3.46 in 2012 (APC: −3.65*, 95% CI: −4.47 to −3.00), but then significantly increased to 3.69 in 2022 (APC: 1.13*, 95% CI: 0.27 to 2.50) (Figure 6; Supplementary Table 5). These represent short-term fluctuations, but the overall trend in this age-group was a decline in NSAH-related mortality.

## Discussion

This study investigates mortality trends of NSAH among young adults aged 15–64 in the United States between 1999 and 2022. The CDC WONDER dataset only provides mortality data related to specific causes of death. Therefore, all potential explanations for trends seen in reported data are hypotheses rather than conclusions. Moreover, due to the use of ICD-10 code I60, changes in relative proportions of aneurysmal vs. non-aneurysmal NSAH could influence observed mortality trends and conclusions.

Key findings in this research study show that the AAMR for NSAH experienced an overall decrease across the study period, with short-term fluctuations contributing to this pattern (AAPC: −1.81*, 95% CI: −2.12 to −1.55) (Supplementary Table 1). This decline in NSAH-related mortality may reflect improved early detection, recent developments in management strategies, and vascular risk factor awareness, although the present study does not assess causal mechanisms (1). However, NSAH-related mortality varies when comparing different demographic groups. The observed overall decrease in AAMR parallels findings seen in current literature. A previous study reports a decline in hospital admissions and case-fatality rates for nontraumatic SAH, largely attributing it to advances in aneurysm detection and treatment (2). Research indicates that implementation of endovascular coiling combined with advanced hypertension management methods may have helped to significantly decrease mortality rates, and further studies should investigate this connection (3).

Despite previous progress, there was a tentatively observed increase in AAMR numbers starting in 2014. Similar trends were noted in a study that reported a rising incidence of stroke in younger adults, attributed to lifestyle-related risk factors such as obesity, metabolic syndrome, as well as substance abuse (4). The slight uptick in AAMR in more recent years could possibly be correlated with an increase in the prevalence of these risk factors, exacerbated by both healthcare access disparities as well as delayed interventions, but this requires further investigation.

During the research period, female study participants continuously displayed higher AAMRs than the male participants. The mortality rates of females dropped more extensively than male rates during this period. Additional studies have already shown that women display increased tendencies toward NSAH development (5). The discrepancy between male and female AAMR rates may be a result of biological factors which regulate vascular function along with estrogen’s impact on arterial walls. The greater overall reduction in female mortality compared to males (AAPC: −2.25% vs. -1.36%) may suggest that interventions, such as controlling blood pressure and strict smoking cessation initiatives, can be particularly effective in the female population. Previous studies on stroke incidence have demonstrated sex-specific patterns that demand targeted prevention strategies based on sex (15). Continued research in sex-specific prevention strategies are necessary to continue the downward mortality trend experienced by women and to continue to lower that of male patients as well.

Our study revealed substantial differences in NSAH-related AAMR between racial groups. The research demonstrates that Black or African American patients and American Indian or Alaska Native patients displayed the highest AAMRs throughout the study period due to documented racial health disparities in stroke literature (16, 17). This is a between-group comparison in NSAH-related mortality based on demographic stratification. The AAMR rate among American Indian or Alaska Native patients experienced a concerning short-term increase following its initial temporary reduction which resulted in no significant total change (AAPC: 0.30). The combination of inadequate healthcare funding and high vascular risk factors and healthcare system obstacles in Indigenous communities could possibly explain these trends (18). The small population size as well as possible limitation of data collection could also contribute to this potential trend. The changing trends toward higher AAMR in Black or African American and Hispanic or Latino groups could possibly indicate increased exposure to avoidable risk elements including substance abuse and hypertension from stress and inadequate healthcare services primarily in economically disadvantaged areas (19). Further studies are indicated to investigate the connection between social risk factors and increased NSAH-related mortality among various racial and ethnic groups.

Regional analysis further reinforces the geographic dimension of health inequity. In the US, geographic disparities in stroke-related mortality are shaped by medical access, risk factor prevalence, as well as numerous historical, socioeconomic, and racial factors. The South’s persistently highest AAMRs correspond with the well-established “Stroke Belt,” an area in the southeastern US with specific cultural, dietary, and socioeconomic factors that contribute to elevated cerebrovascular mortality (9, 20). Notably, the South was the only region to experience a significant short-term increase in AAMR between 2015 and 2020, a pattern possibly exacerbated by uneven access to specialized stroke centers and preventive care (21). These findings underscore the necessity of targeted, community-level interventions to mitigate rising risks in already vulnerable populations.

The overall AAMR numbers temporarily decreased between 2015 and 2020 in both rural and urban areas, but urban areas demonstrated a steadier downward trend throughout the entire study period. The data from rural areas showed inconsistent results due to a tentative rise in mortality between 2017 and 2020. This may be influenced by potential barriers in healthcare delivery, emergency response times, and specialist availability in rural areas in the US. These findings demonstrate support for previous research emphasizing how geographic variations impact stroke results and require localized public policy measures for substantial improvements but further investigate is needed to corroborate these hypotheses (22).

Considering age-specific trends, the crude mortality rate for NSAH declined across all 10-year age groups (15–24, 25–34, 35–44, 45–54, and 55–64) from 1999 to 2022. The age group spanning 55 to 64 years old maintained the highest mortality rates throughout the study period while the 15 to 24 age group maintained the lowest rates. The mortality rates decreased consistently for all age groups, but the 45–54 and 55–64 age groups were observed to experience temporary increases between 2020 and 2021. These fluctuations may reflect age-related cumulative exposure to vascular risk factors such as hypertension, smoking, and alcohol use, which are known contributors to NSAH mortality in older adults (5, 23). Moreover, delayed healthcare engagement during the COVID-19 pandemic may have further contributed to these short-term fluctuations increases in the years 2020–2021 (24).

## Conclusion

The observed overall decrease in NSAH-related mortality in young adults likely reflects advancements in neurosurgical care, vascular risk management, and aneurysm detection. However, persistent disparities across sex and, more notably, race and region underscore the potential influence of systemic factors, including access to healthcare, socioeconomic status, and prevalence of risk factors like hypertension, smoking, and alcohol use. Rising mortality rates in particular racial groups and the Southern census region highlight a need for targeted public health interventions. Knowledge of these temporal patterns and population demographics in NSAH mortality is critical to development of prevention measures, early detection, and post-bleeding care practices. These research findings show the necessity for continued monitoring of NSAH trends to guide public health care efforts and stroke prevention initiatives in younger adult populations.

### Limitations

1. Analysis relies on mortality data driven from the CDC WONDER database, which is based on death certificates. The accuracy of cause-of-death reporting on death certificates can vary because of misclassification or underreporting of contributing factors.
2. This study is focused on young adults aged 15 to 64 years. Observed mortality trends cannot be generalizable for older populations/children/adolescents outside this specific age range.
3. Racial and ethnicity data are grouped into broad categories (White, Black or African American, Asian or Pacific Islander, and Hispanic or Latino), which may mask important variations or heterogeneity within these groups.
4. As noted in the methods and results section, data for certain states and specific demographic strata may be marked as unreliable, due to small values that are suppressed by the CDC WONDER database. This potentially limits the statistical power of analyses for these subgroups.
5. This study is an ecological analysis of population-level mortality data. As such, it can identify trends and disparities at a group level but cannot establish individual-level risk factors or causal relationships for the observed mortality patterns. Factors such as socioeconomic status, access to specialized medical care, and individual lifestyle choices, which are known to influence stroke risk and outcomes, could not be directly assessed using this database.
6. Beyond this, due to limitations of available research and data, some possible explanations for trends are based on sources more than 10 years old. Although these are still valid sources and important points to make in this paper, certain areas of the literature could benefit from updates.

## Data Availability

Publicly available datasets were analyzed in this study. This data can be found at: https://wonder.cdc.gov/.
