# Supplementary material for: Trends in nontraumatic subarachnoid hemorrhage-related mortality among young adult (15–64 years) population in the United States, 1999–2022
Source: Front Neurol. 2025 Oct 3;16:1646709. doi: 10.3389/fneur.2025.1646709 (PMC12531071; doi:10.3389/fneur.2025.1646709)
Supplement: Supplementary file 1 [file Datasheet_1.docx]

**Supplemental Figures and Tables.**

**Supplemental Figure 1.** Trends in Overall NSAH-Related Age-Adjusted Mortality Rates (AAMR) and Stratified by Gender in the US, 1999 to 2022

- **Overall**
  - 1999 to 2014 APC: -3.53*
  - 2014 to 2022 APC: 1.50*
- **Female**
  - 1999 to 2014 APC: -4.02*
  - 2014 to 2022 APC: 1.15
- **Male**
  - 1999 to 2013 APC: -2.94*
  - 2013 to 2022 APC: 1.16*


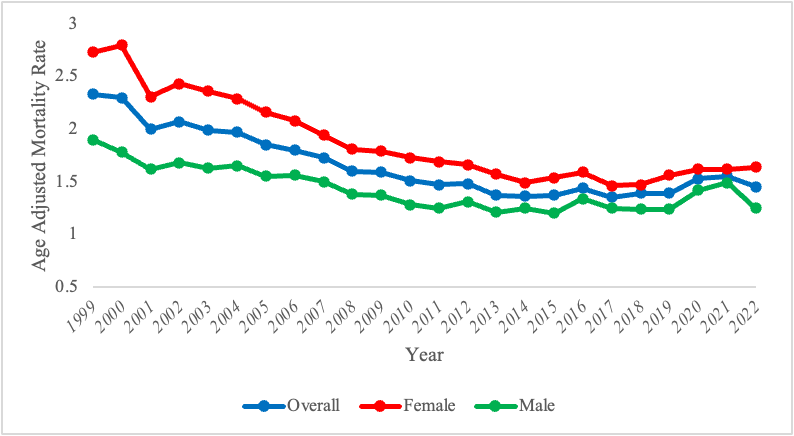


**Supplemental Table 1.** Overall NSAH Mortality Trends and Stratified by Gender.

|  | Age-Adjusted Mortality Rate | | |
| --- | --- | --- | --- |
| Year | Overall | Female | Male |
| 1999 | 2.33 | 2.73 | 1.9 |
| 2000 | 2.3 | 2.8 | 1.78 |
| 2001 | 2 | 2.31 | 1.62 |
| 2002 | 2.07 | 2.43 | 1.68 |
| 2003 | 1.99 | 2.36 | 1.63 |
| 2004 | 1.97 | 2.29 | 1.65 |
| 2005 | 1.85 | 2.16 | 1.55 |
| 2006 | 1.8 | 2.08 | 1.56 |
| 2007 | 1.73 | 1.94 | 1.5 |
| 2008 | 1.6 | 1.81 | 1.38 |
| 2009 | 1.59 | 1.79 | 1.37 |
| 2010 | 1.51 | 1.73 | 1.28 |
| 2011 | 1.47 | 1.69 | 1.25 |
| 2012 | 1.48 | 1.66 | 1.31 |
| 2013 | 1.37 | 1.57 | 1.21 |
| 2014 | 1.36 | 1.49 | 1.25 |
| 2015 | 1.37 | 1.54 | 1.2 |
| 2016 | 1.44 | 1.59 | 1.34 |
| 2017 | 1.35 | 1.46 | 1.25 |
| 2018 | 1.39 | 1.47 | 1.24 |
| 2019 | 1.39 | 1.56 | 1.24 |
| 2020 | 1.53 | 1.62 | 1.42 |
| 2021 | 1.55 | 1.62 | 1.49 |
| 2022 | 1.45 | 1.64 | 1.25 |
| Number of Joinpoints (Years of Joinpoint) | 1 (2014) | 1 (2014) | 1 (2013) |
| APC Segment 1 (95% CI) | -3.53* | -4.02* | -2.94 |
| APC Segment 2 (95% CI) | -1.50* | 1.15 | 1.16 |
| Average APC (95% CI) | -1.81* | -2.25* | -1.36* |

**Supplemental Figure 2.** Trends in NSAH-Related Age-Adjusted Mortality Rates (AAMR) and Stratified by Race in the US, 1999 to 2022

- **American Indian**
  - 1999 to 2018 APC: -2.03*
  - 2018 to 2022 APC: 12.16*
- **Asian or Pacific Islander**
  - 1999 to 2014 APC: -2.67*
  - 2014 to 2022 APC: 0.15
- **Black or African American**
  - 1999 to 2015 APC: -4.40*
  - 2015 to 2022 APC: 2.05*
- **White**
  - 1999 to 2013 APC: -3.71*
  - 2013 to 2022 APC: 0.52
- **Hispanic or Latino**
  - 1999 to 2015 APC: -3.29*
  - 2015 to 2022 APC: 2.30*


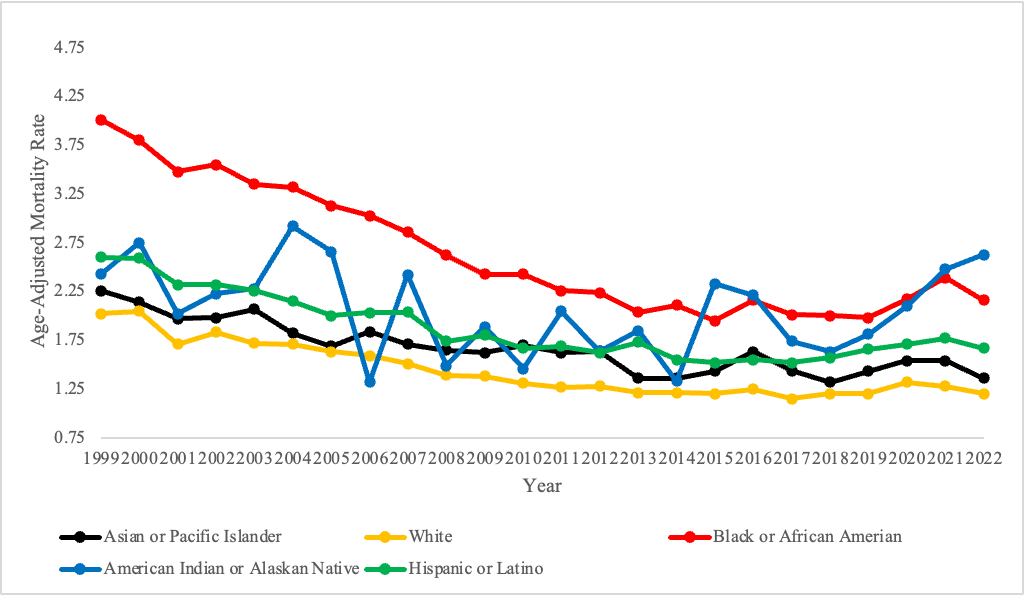


**Supplemental Table 2.** NSAH Mortality Trends Stratified by Race.

|  | Age-Adjusted Mortality Rate | | | | |
| --- | --- | --- | --- | --- | --- |
| Year | American Indian or Alaska Native | Asian or Pacific Islander | Black or African American | White | Hispanic or Latino |
| 1999 | 2.43 | 2.26 | 4.01 | 2.02 | 2.6 |
| 2000 | 2.75 | 2.14 | 3.8 | 2.05 | 2.59 |
| 2001 | 2.02 | 1.97 | 3.48 | 1.71 | 2.32 |
| 2002 | 2.23 | 1.98 | 3.55 | 1.83 | 2.32 |
| 2003 | 2.28 | 2.07 | 3.35 | 1.72 | 2.26 |
| 2004 | 2.92 | 1.82 | 3.32 | 1.71 | 2.15 |
| 2005 | 2.66 | 1.69 | 3.13 | 1.63 | 2 |
| 2006 | 1.32 | 1.84 | 3.03 | 1.59 | 2.03 |
| 2007 | 2.42 | 1.71 | 2.86 | 1.51 | 2.04 |
| 2008 | 1.49 | 1.65 | 2.62 | 1.39 | 1.74 |
| 2009 | 1.89 | 1.62 | 2.43 | 1.38 | 1.8 |
| 2010 | 1.46 | 1.7 | 2.43 | 1.31 | 1.67 |
| 2011 | 2.05 | 1.62 | 2.26 | 1.27 | 1.69 |
| 2012 | 1.64 | 1.64 | 2.24 | 1.28 | 1.62 |
| 2013 | 1.85 | 1.36 | 2.04 | 1.21 | 1.73 |
| 2014 | 1.33 | 1.36 | 2.11 | 1.21 | 1.55 |
| 2015 | 2.33 | 1.43 | 1.95 | 1.2 | 1.52 |
| 2016 | 2.21 | 1.63 | 2.16 | 1.25 | 1.55 |
| 2017 | 1.74 | 1.44 | 2.01 | 1.15 | 1.52 |
| 2018 | 1.63 | 1.32 | 2 | 1.2 | 1.57 |
| 2019 | 1.81 | 1.43 | 1.98 | 1.2 | 1.66 |
| 2020 | 2.1 | 1.54 | 2.17 | 1.32 | 1.71 |
| 2021 | 2.48 | 1.54 | 2.39 | 1.28 | 1.77 |
| 2022 | 2.63 | 1.36 | 2.16 | 1.2 | 1.67 |
| Number of Joinpoints (Years of Joinpoint) | 1 (2018) | 1 (2014) | 1 (2015) | 1 (2013) | 1 (2015) |
| APC Segment 1 (95% CI) | -2.03* | -2.67* | -4.40* | -3.71* | -3.29* |
| APC Segment 2 (95% CI) | 12.16 | 0.15 | 2.05* | 0.52 | 2.30* |
| Average APC (95% CI) | 0.30 | -1.69* | -2.48* | -2.07* | -1.62* |

**Supplemental Figure 3.** Trends in NSAH-Related Age-Adjusted Mortality Rates (AAMR) and Stratified by US Census Region, 1999 to 2022

- **Northeast**
  - 1999 to 2012 APC: -3.79*
  - 2012 to 2020 APC: -0.82
- **Midwest**
  - 1999 to 2013 APC: -3.75*
  - 2013 to 2020 APC: -0.38
- **South**
  - 1999 to 2015 APC: -3.58*
  - 2015 to 2020 APC: -3.38*
- **West**
  - 1999 to 2011 APC: -3.43*
  - 2011 to 2020 APC: 0.07


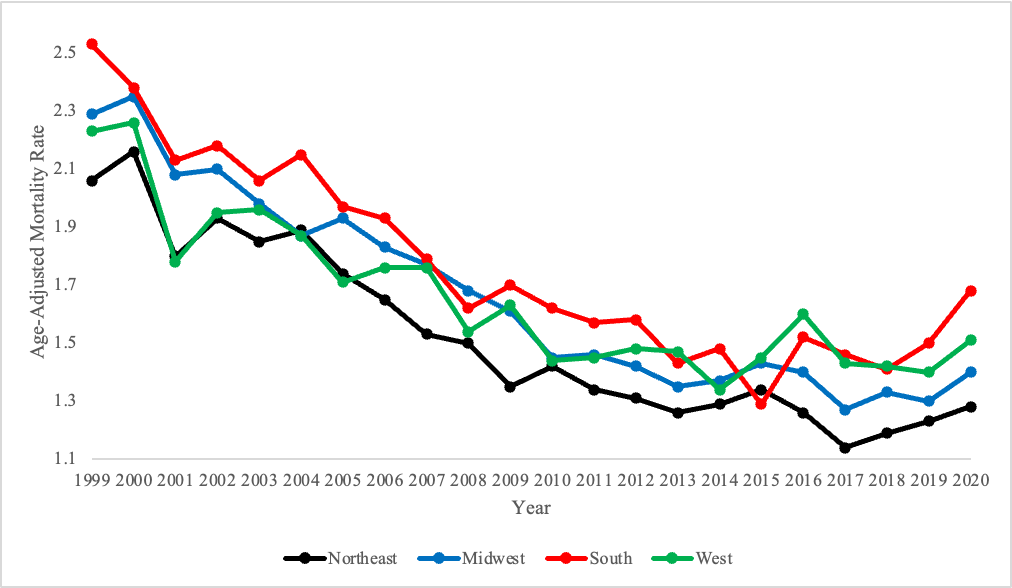


**Supplemental Table 3.** NSAH Mortality Trends Stratified by US Census Region.

|  | Age-Adjusted Mortality Rate | | | |
| --- | --- | --- | --- | --- |
| Year | Northeast | Midwest | South | West |
| 1999 | 2.06 | 2.29 | 2.53 | 2.23 |
| 2000 | 2.16 | 2.35 | 2.38 | 2.26 |
| 2001 | 1.8 | 2.08 | 2.13 | 1.78 |
| 2002 | 1.93 | 2.1 | 2.18 | 1.95 |
| 2003 | 1.85 | 1.98 | 2.06 | 1.96 |
| 2004 | 1.89 | 1.87 | 2.15 | 1.87 |
| 2005 | 1.74 | 1.93 | 1.97 | 1.71 |
| 2006 | 1.65 | 1.83 | 1.93 | 1.76 |
| 2007 | 1.53 | 1.77 | 1.79 | 1.76 |
| 2008 | 1.5 | 1.68 | 1.62 | 1.54 |
| 2009 | 1.35 | 1.61 | 1.7 | 1.63 |
| 2010 | 1.42 | 1.45 | 1.62 | 1.44 |
| 2011 | 1.34 | 1.46 | 1.57 | 1.45 |
| 2012 | 1.31 | 1.42 | 1.58 | 1.48 |
| 2013 | 1.26 | 1.35 | 1.43 | 1.47 |
| 2014 | 1.29 | 1.37 | 1.48 | 1.34 |
| 2015 | 1.34 | 1.43 | 1.29 | 1.45 |
| 2016 | 1.26 | 1.4 | 1.52 | 1.6 |
| 2017 | 1.14 | 1.27 | 1.46 | 1.43 |
| 2018 | 1.19 | 1.33 | 1.41 | 1.42 |
| 2019 | 1.23 | 1.3 | 1.5 | 1.4 |
| 2020 | 1.28 | 1.4 | 1.68 | 1.51 |
| Number of Joinpoints (Years of Joinpoint) | 1 (2012) | 1 (2013) | 1 (2015) | 1 (2011) |
| APC Segment 1 (95% CI) | -3.79* | -3.75* | -3.58* | -3.43* |
| APC Segment 2 (95% CI) | -0.82 | -0.38 | 3.38* | 0.07 |
| Average APC (95% CI) | -2.67* | -2.64* | -1.96* | -1.94* |

**Supplemental Figure 4.** Trends in NSAH-Related Age-Adjusted Mortality Rates (AAMR) Stratified by State, 1999-2020.


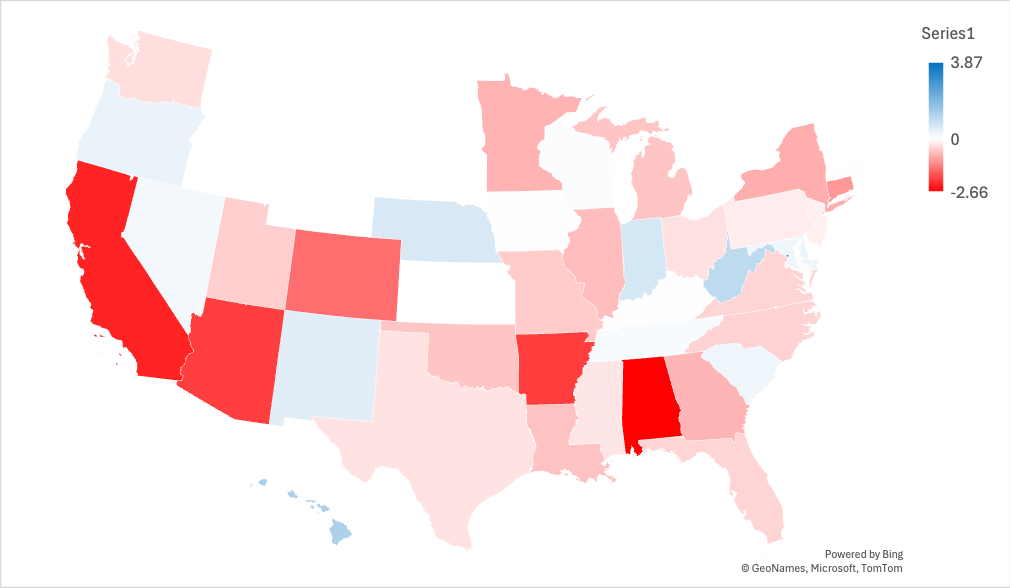


**Supplemental Figure 5.** Trends in NSAH-Related Age-Adjusted Mortality Rates (AAMR) Stratified by State, 2020-2022.


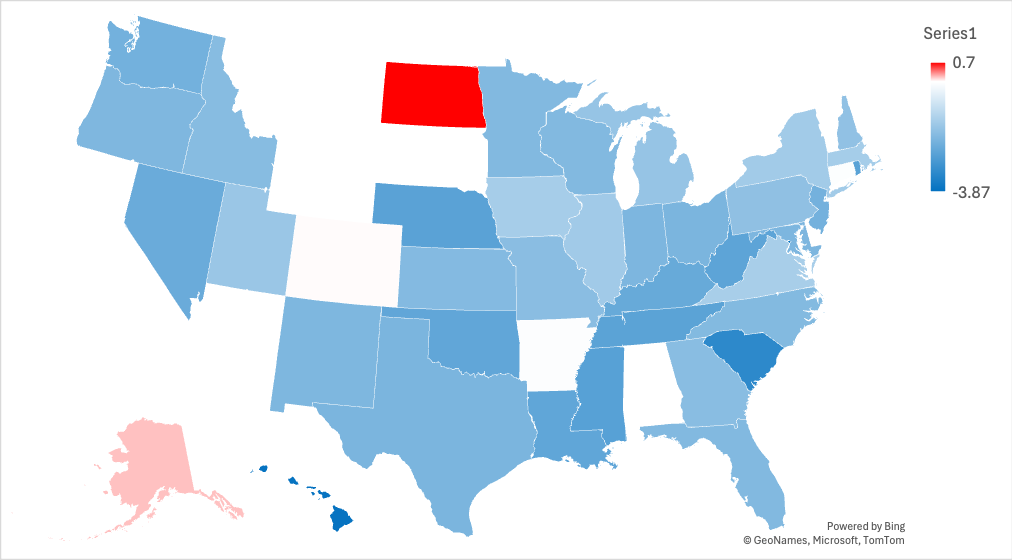


**Supplemental Figure 6.** Trends in NSAH-Related Age-Adjusted Mortality Rates (AAMR) and Stratified by Rural vs. Urban Classifications, 1999 to 2022

- **Rural**
  - 1999 to 2012 APC: -2.94
  - 2012 to 2018 APC: -0.59
  - 2018 to 2020 APC: 10.48*
- **Urban**
  - 1999 to 2013 APC: -3.78*
  - 2013 to 2020 APC: 0.39


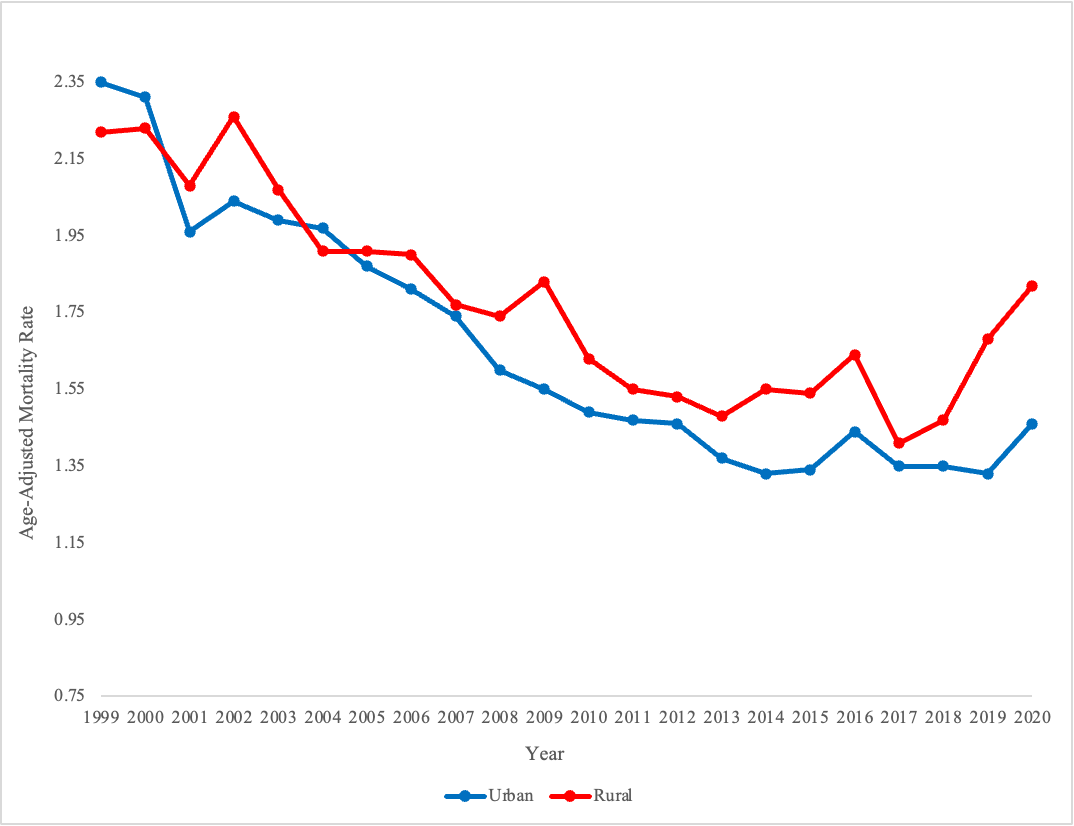


**Supplemental Table 4.** NSAH Mortality Trends Stratified by Rural vs. Urban Classification.

|  | Age-Adjusted Mortality Rate | |
| --- | --- | --- |
| Year | Rural | Urban |
| 1999 | 2.22 | 2.35 |
| 2000 | 2.23 | 2.31 |
| 2001 | 2.08 | 1.96 |
| 2002 | 2.26 | 2.04 |
| 2003 | 2.07 | 1.99 |
| 2004 | 1.91 | 1.97 |
| 2005 | 1.91 | 1.87 |
| 2006 | 1.9 | 1.81 |
| 2007 | 1.77 | 1.74 |
| 2008 | 1.74 | 1.6 |
| 2009 | 1.83 | 1.55 |
| 2010 | 1.63 | 1.49 |
| 2011 | 1.55 | 1.47 |
| 2012 | 1.53 | 1.46 |
| 2013 | 1.48 | 1.37 |
| 2014 | 1.55 | 1.33 |
| 2015 | 1.54 | 1.34 |
| 2016 | 1.64 | 1.44 |
| 2017 | 1.41 | 1.35 |
| 2018 | 1.47 | 1.35 |
| 2019 | 1.68 | 1.33 |
| 2020 | 1.82 | 1.46 |
| Number of Joinpoints (Years of Joinpoint) | 2 (2012, 2018) | 1 (2013) |
| APC Segment 1 (95% CI) | -2.94 | -3.78* |
| APC Segment 2 (95% CI) | -0.59 | 0.39 |
| APC Segment 3 (95% CI) | 10.48* | - |
| Average APC (95% CI) | -1.06* | -2.41* |

**Supplemental Figure 7.** Trends in NSAH-Related Crude Mortality Rate and Stratified by Age, 1999 to 2022

- **15-24 years**
  - 1999 to 2022 APC: -2.01*
- **25-34 years**
  - 1999 to 2014 APC: -2.83*
  - 2014 to 2022 APC: 1.33
- **35-44 years**
  - 1999 to 2013 APC: -4.37*
  - 2013 to 2022 APC: 0.49
- **45-54 years**
  - 1999 to 2015 APC: -3.23*
  - 2015 to 2022 APC: 1.27
- **55-64 years**
  - 1999 to 2012 APC: -3.65*
  - 2012 to 2022 APC: 1.13*


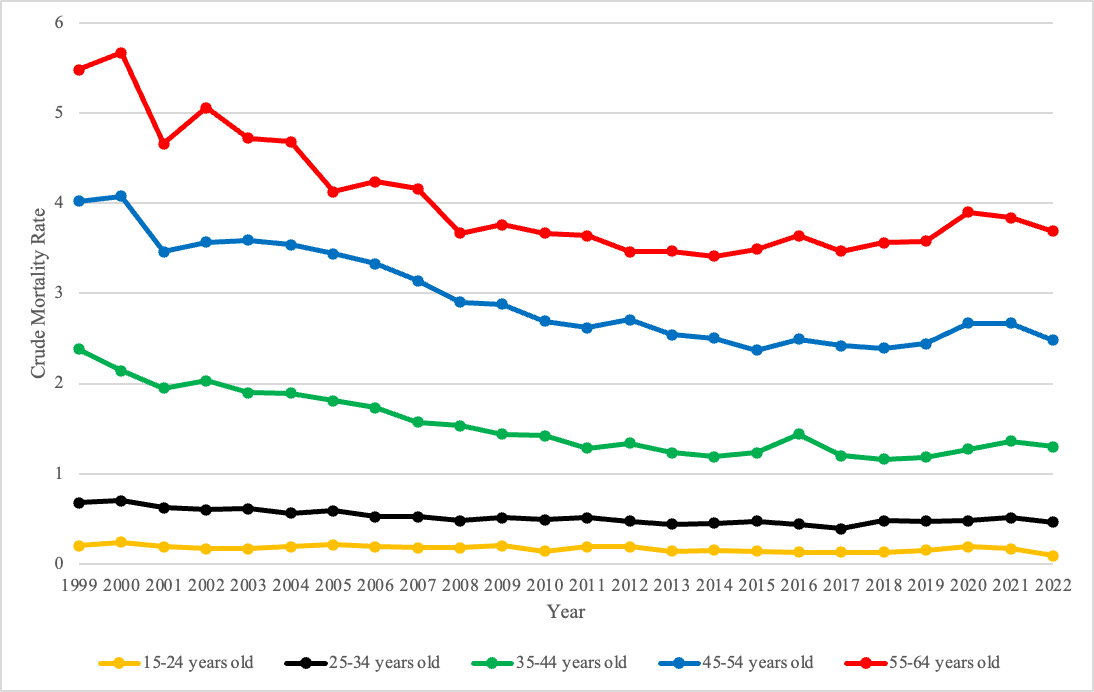


**Supplemental Table 5.** NSAH Mortality Trends Stratified by 10-Year Age Group.

|  | Crude Mortality Rate | | | | |
| --- | --- | --- | --- | --- | --- |
| Year | 15-24 years | 25-34 years | 35-44 years | 45-54 years | 55-64 years |
| 1999 | 0.2 | 0.68 | 2.38 | 4.02 | 5.48 |
| 2000 | 0.24 | 0.7 | 2.14 | 4.08 | 5.67 |
| 2001 | 0.19 | 0.62 | 1.95 | 3.46 | 4.66 |
| 2002 | 0.17 | 0.6 | 2.03 | 3.57 | 5.06 |
| 2003 | 0.17 | 0.61 | 1.9 | 3.59 | 4.72 |
| 2004 | 0.19 | 0.56 | 1.89 | 3.54 | 4.68 |
| 2005 | 0.21 | 0.59 | 1.81 | 3.44 | 4.13 |
| 2006 | 0.19 | 0.52 | 1.73 | 3.33 | 4.24 |
| 2007 | 0.18 | 0.52 | 1.57 | 3.14 | 4.16 |
| 2008 | 0.18 | 0.48 | 1.53 | 2.9 | 3.67 |
| 2009 | 0.2 | 0.51 | 1.44 | 2.88 | 3.76 |
| 2010 | 0.14 | 0.49 | 1.42 | 2.69 | 3.67 |
| 2011 | 0.19 | 0.51 | 1.28 | 2.62 | 3.64 |
| 2012 | 0.19 | 0.47 | 1.34 | 2.71 | 3.46 |
| 2013 | 0.14 | 0.44 | 1.23 | 2.54 | 3.47 |
| 2014 | 0.15 | 0.45 | 1.19 | 2.5 | 3.41 |
| 2015 | 0.14 | 0.47 | 1.23 | 2.37 | 3.49 |
| 2016 | 0.13 | 0.44 | 1.44 | 2.49 | 3.64 |
| 2017 | 0.13 | 0.39 | 1.2 | 2.42 | 3.47 |
| 2018 | 0.13 | 0.48 | 1.16 | 2.39 | 3.56 |
| 2019 | 0.15 | 0.47 | 1.18 | 2.44 | 3.58 |
| 2020 | 0.19 | 0.48 | 1.27 | 2.67 | 3.9 |
| 2021 | 0.17 | 0.51 | 1.36 | 2.67 | 3.84 |
| 2022 | 0.09 | 0.46 | 1.3 | 2.48 | 3.69 |
| Number of Joinpoints (Years of Joinpoint) | 0 | 1 (2014) | 1 (2013) | 1 (2015) | 1 (2015) |
| APC Segment 1 (95% CI) | -2.01* | -2.83* | -4.37* | -3.23* | -3.65* |
| APC Segment 2 (95% CI) | - | 1.33 | 0.49 | 1.27 | 1.13* |
| Average APC (95% CI) | -2.01* | -1.41* | -2.50* | -1.88* | -1.60* |
